# Health risk assessment of rare earth elements in cereals from mining area in Shandong, China

**DOI:** 10.1038/s41598-017-10256-7

**Published:** 2017-08-29

**Authors:** Maoqiang Zhuang, Liansen Wang, Guangjian Wu, Kebo Wang, Xiaofeng Jiang, Taibin Liu, Peirui Xiao, Lianlong Yu, Ying Jiang, Jian Song, Junli Zhang, Jingyang Zhou, Jinshan Zhao, Zunhua Chu

**Affiliations:** 1 Shandong Center for Disease Control and Prevention, Jinan, Shandong P.R. China; 2 Shandong Center for Food Safety Risk Assessment, Jinan, Shandong P.R. China; 3 Academy of Preventive Medicine, Shandong University, Jinan, Shandong P.R. China; 4 Qilu Hospital of Shandong University, Jinan, Shandong P.R. China; 5 Laiwu Municipal Center for Disease Control and Prevention, Laiwu, Shandong P.R. China

## Abstract

To investigate the concentrations of rare earth elements in cereals and assess human health risk through cereal consumption, a total of 327 cereal samples were collected from rare earth mining area and control area in Shandong, China. The contents of 14 rare earth elements were determined by Inductively Coupled Plasma—Mass Spectrometry (ICP—MS). The medians of total rare earth elements in cereals from mining and control areas were 74.22 μg/kg and 47.83 μg/kg, respectively, and the difference was statistically significant (*P* < 0.05). The wheat had the highest rare earth elements concentrations (109.39 μg/kg and 77.96 μg/kg for mining and control areas, respectively) and maize had the lowest rare earth elements concentrations (42.88 μg/kg and 30.25 μg/kg for mining and control areas, respectively). The rare earth elements distribution patterns for both areas were characterized by enrichment of light rare earth elements. The health risk assessment demonstrated that the estimated daily intakes of rare earth elements through cereal consumption were considerably lower than the acceptable daily intake (70 μg/kg bw). The damage to adults can be neglected, but more attention should be paid to the effects of continuous exposure to rare earth elements on children.

## Introduction

The rare earth elements (REE) include seventeen chemical elements: fifteen lanthanides [lanthanum (La), cerium (Ce), praseodymium (Pr), neodymium (Nd), promethium (Pm), samarium (Sm), europium (Eu), gadolinium (Gd), terbium (Tb), dysprosium (Dy), holmium (Ho), erbium (Er), thulium (Tm), ytterbium (Yb), lutetium (Lu)] and scandium (Sc) and yttrium (Y). Sc and Y are considered REE because they tend to exist in the same ore deposits with the lanthanides and exhibit similar chemical properties. La, Ce, Pr, Nd, Sm, Eu are also indicated as light REE because of their atomic mass lower than 153 while Gd, Tb, Dy, Ho, Er, Tm, Yb and Lu are also indicated as heavy REE because of their atomic mass greater than 153.

REE have been utilized in a number of industrial, medical and agricultural or zootechnical applications due to their specific properties^1–5^. It is estimated that the REE enriched fertilizers released into the cultivated soil were 5,200 tons in 2002 in China^6^. In addition, large-scale exploitation activities of REE resources have resulted in substantial increase of the contamination levels in soil and water around the mining area^7–11^. Huang *et al*.^12^ estimated that rare earth oxides entering into the soil due to mining activities with low extraction rate of 50% were 119,000 tons in 2005 in China. For these reasons, people are increasingly interested in the bioaccumulation and health risk of REE^13–16^. It has been listed as “New and Emerging Risks to Occupational Safety and Health” by the European Agency for Safety and Health at Work^17^. REE in soil and water are released and partly enter human body through multiple exposure pathways, especially food ingestion. As nonessential elements in organisms, the effects of REE accumulation on organisms remain fragmentary and inconsistent. Furthermore, the toxicological mechanisms and related environmental risk remain unclear^18^. Although there is no report on incidents of human poisoning through food chain, potential concerns regarding effects of continuous exposure to low levels of REE on human health have been arising because they are accumulated in blood, brain and bone after entering human body and long-term exposure to REE may be related to health problems such as changes in brain and bone^19–23^. Besides these, people are increasingly interested in the impact of REE on children’s neurodevelopment. Researches for children showed that REE might be related to decreased IQ and memory loss^24–26^. It is therefore necessary to investigate their concentration levels in daily food of vegetable, grain and meat to assess the potential risk of REE to human health.

The rare earth ore in present study is located in Weishan County in the southwest of Shandong province and is one of the three largest light rare earth ores in China. The abundances of lanthanum, cerium, praseodymium and neodymium account for more than 98% of the total REE.

This is the first study dealing with REE in cereals of local households in close proximity to a large-scale rare earth mining site in Shandong, China. The main objectives were: (1) to investigate the levels of REE in cereals of mining and control areas in Weishan County; (2) to evaluate the health risk of dietary REE exposure through cereal consumption.

## Results and Discussion

### REE levels in cereals

The average concentration of REE for all 327 samples was 55.79 μg/kg. For cereals collected from mining and control areas, the average concentrations were 74.22 μg/kg and 47.83 μg/kg, respectively, and La, Ce, Pr and Nd were major elements and accounted for over 90% of total REE for mining area (Table 1). The total REE and light REE were statistically significant different between cereals from mining and control areas (*P < *0.05). The results are consistent with previous studies, but the REE levels in the present study are relatively lower^27, 28^.

**Table 1:** REE concentrations in cereals from mining and control areas (μg/kg).

Element	Mining area		Control area		Total	
	Median	IQR	Median	IQR	Median	IQR
La	23.01	52.43	19.07	39.60	20.05	43.23
Ce	31.70	102.72	12.49	25.45	18.05	46.99
Pr	2.78	9.03	1.59	3.09	1.83	4.43
Nd	9.04	30.79	5.28	11.15	6.25	15.67
Sm	1.41	3.99	1.03	2.22	1.17	2.83
Eu	1.21	1.66	0.47	1.47	0.99	1.61
Gd	1.52	4.81	1.19	2.36	1.27	3.04
Tb	0.16	0.40	0.13	0.27	0.14	0.32
Dy	0.66	1.71	0.64	1.27	0.65	1.42
Ho	0.12	0.29	0.11	0.23	0.11	0.25
Er	0.37	0.94	0.35	0.66	0.36	0.75
Tm	0.03	0.10	0.04	0.10	0.04	0.10
Yb	0.24	0.63	0.30	0.61	0.27	0.63
Lu	0.04	0.06	0.04	0.07	0.04	0.07
LREE	70.53	202.34	46.51	77.88	53.05	111.91
HREE	3.13	7.82	2.74	5.51	2.90	6.47
ΣREE	74.22	216.81	47.83	81.74	55.79	115.87

Abbreviations: IQR: interquartile range, LREE: light rare earth elements, HREE: heavy rare earth elements, ΣREE: total rare earth elements.

The reasons for the differences in REE levels between different studies include the type of rare earth ore, REE levels in soils, and plant species. High concentration level of REE in soil can lead to more absorption and accumulation of REE^29^. Plant uptake of REE also depends on mobility and bioavailability of REE in soil^30, 31^. Different type of rare earth ore has different mobility and bioavailability of REE influencing the absorption of REE.

### REE levels in different categories of cereals

We divided the samples into three categories: wheat, maize and legume. For both mining and control areas, The REE concentrations of wheat were 150% higher than that of maize. The maize from mining area had significant higher REE concentrations than control area (*P* = 0.03). For wheat and legume, there was no statistically significant difference in REE concentrations between mining and control area (Table 2). For both mining and control areas, the REE concentrations in cereals declined in the order of wheat > legume > maize. Previous studies had demonstrated the similar result^27, 28^. Results of a study on REE absorption and distribution showed that the maize roots had much higher REE concentrations than other parts of maize. Further analysis demonstrated that REE were closely bound to the cell walls in the root cells^32^. This may be the reason that maize has the lowest REE levels compared to wheat and legume.

**Table 2:** Total REE concentrations in cereals from mining and control areas (μg/kg).

Category	Mining area			Control area			Z	*P*
	N	Median	IQR	N	Median	IQR		
wheat	87	109.39	338.69	53	77.96	145.65	−0.68	0.49
maize	57	42.88	98.44	83	30.25	54.12	2.23	0.03
legume	25	95.09	120.71	22	58.46	93.36	1.25	0.21

Abbreviations: IQR: interquartile range.

### REE distribution pattern

**Figure 1:**
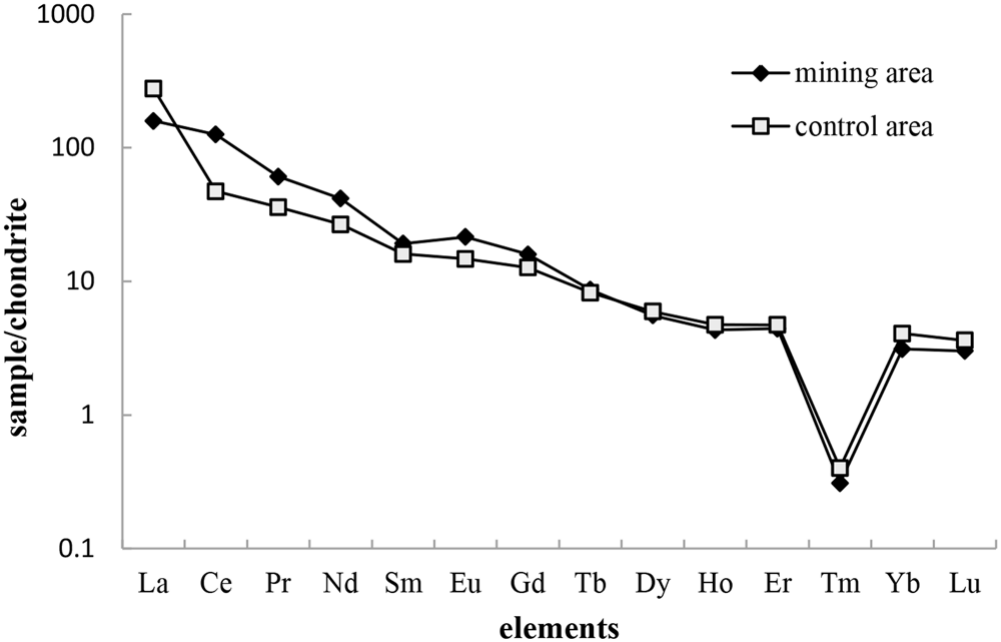
Chondrite-normalized REE distribution patterns for cereals. The REE abundances were normalized to those in chondrite, and then the pattern was achieved by plotting the ratios on a logarithmic scale against the atomic number. The REE in chondrite were assumed to be no fractionation. This could eliminate the abundant changes between odd and even atomic numbers.

The ratio of light REE to heavy REE (22.36 for cereals from mining area and 18.48 for control area) and the apparent negative slope in Fig. 1 showed that the total REE was dominated by light REE. The values of δEu and δCe of mining area and value of δEu of control area were close to 1, but the value of δCe of control area indicated that there was an pronounced Ce anomaly (Table 3).

**Table 3:** REE characteristic values in cereals from mining and control areas.

Area	L/H	δEu	δCe	(La/Yb)_N_	(La/Sm)_N_	(Gd/Yb)_N_
Mining area	22.36	1.23	1.28	50.88	8.27	5.11
Control area	18.48	1.03	0.47	67.89	17.25	3.11

Rare earth elements were Chondrite-normalized by W.V. Boynton (1984) recommended chondrite abundance; L/H: light REE/heavy REE; (La/Sm)N and (Gd/Yb)N mean the internal differentiation status of LREE and HREE, respectively; δEu, and δCe mean abnormality degree of Ce, and Eu. The subscript N refers to the relative abundance after chondrite was standardized: δCe = CeN/(LaN × PrN)0.5, δEu = EuN/(SmN × GdN)0.5.

Although the REE abundances of mining and control areas were different, the REE distribution patterns in cereals were consistent. This may be due to the fractionation of light REE and heavy REE. (La/Sm)_N_ and (Gd/Yb)_N_ indicated that the internal differentiation status of light REE and heavy REE was moderate. However, for control area, the internal differentiation status of light REE was higher than mining area. In addition, there was an obvious Tm anomaly in the distribution patterns. We think the reason might be the low Tm abundance in samples, which is far less than that in chondrites, resulting in a very low ratio of sample/chondrite.

### Human health risk assessment

For different gender/age groups, the estimated daily intakes (EDI) of the total rare earth oxides were considerably lower than the established allowable daily intake (70 μg/kg bw), even calculated with 95% quantile of rare earth oxides in cereals, but children aged 2–12 years had higher EDI than the other group, especially the group of 2–7 years. The EDI of people over 13 years were substantially the same and had little variation (Table 4).

**Table 4:** Estimated daily intake (μg/kg bw) of total rare earth oxides via cereal consumption in mining and control areas by different gender/age groups.

Gender/age group	BW^a^	CR^a^	Mining area		Control area	
			EDI^b^	EDI^c^	EDI^b^	EDI^c^
2–7 years old	17.9	251.0	1.24	14.36	0.80	8.88
8–12 years old	33.1	400.5	1.07	12.39	0.69	7.67
Male, 13–19 years old	56.4	567.6	0.89	10.31	0.57	6.38
Female, 13–19 years old	50.0	462.4	0.82	9.47	0.53	5.86
Male, 20–50 years old	63.0	587.3	0.82	9.55	0.53	5.91
Female, 20–50 years old	56.0	497.8	0.79	9.10	0.51	5.63
Male, 51–65 years old	65.0	590.6	0.80	9.31	0.52	5.76
Female, 51–65 years old	58.0	501.2	0.76	8.85	0.49	5.47
Male, >65 years old	59.5	512.9	0.76	8.83	0.49	5.46
Female, >65 years old	52.0	405.3	0.69	7.98	0.44	4.94

Abbreviations: BW: body weight, CR: consumption rate, EDI: estimated daily intake.

^a^Data were from the fourth China total diet study.

^b^Calculated with median of rare earth oxides in cereal.

^c^Calculated with 95% quantile of rare earth oxides in cereal.

Based on these results, the harm of REE exposure to adults through the consumption of these cereals is negligible. A study conducted in Fujian province also demonstrated that vegetable consumption would not result in exceeding the safe value of REE EDI for adults^33^. However, it is worth noting that children’s neurodevelopment is more susceptible to REE that is related to decreased IQ and memory loss. Another paper also found that children had higher REE intake through vegetable consumption than adults^34^. So more attention should be paid to the effects of continuous exposure to low levels of REE on children’s nervous system.

It should be noted that the health risk assessment results might be influenced by other factors such as other food (vegetables, meats, and fruits) ingestion and bioavailability of REE. The REE intake through dermal absorption and breath inhalation was not estimated. In addition, the data of consumption rate was obtained in 2007 and may have been changed after these years of economic development. Therefore, a more systematic risk assessment is needed.

## Materials and Methods

### Sampling and pretreatment

The samples were collected from 10 sampling sites (5 from mining area and 5 from control area) during July and October in 2014. The mining area is located in the vicinity of Weishan rare earth ore and the control area was chosen from the site located 70 km away from the mining area. The living conditions, economic backgrounds and cultural and living habits are similar between the mining area and the control area. The natural environment is not affected by the rare earth mining area. The cereals were grown by the local residents and stored at house. Each sample was asked whether it was grown or bought in the market. The samples bought in the market were excluded. A total of 327 samples (edible part) including wheat, maize and legume were collected. Samples were washed with tap water and further washed three times with deionized water. Then, the samples were ground into powder in an agate mortar, passed through 0.149-mm nylon sieve, and stored at −20 °C until analysis was made.

### Sample analysis

All samples were analyzed for REE using procedures established by CFSA^35^. The microwave digestion system was used for analysis where approximately 0.50 g of the sample was weighed and digested with 8 ml of concentrated nitric acid (65%) in a PTFE digestion vessel. The solution was then poured into a volumetric flask and diluted to 10 ml with ultra-high purity water after cooling for about 40 min. 14 REE (excluding Sc, Pm, and Y) were determined using inductively coupled plasma mass spectrometry (Thermo iCAP Q, Thermo Fisher Scientific, Bremen, Germany). REE concentration was expressed as μg/kg. External calibration was performed by measuring standard solutions obtained from national center of analysis and testing for nonferrous metals and electronic materials, China (NCATN). The standard solutions contained these 14 REE at 0, 0.05, 0.1, 0.5, 1, and 10 μg/L levels. Mixed solutions containing Rh, In, and Re obtained from NCATN were used as on-line internal standard at 1 μg/L level. The limits of detection (LOD) of these 14 REE for this method were 0.03–1.02 μg/kg, which were determined as three times of standard deviation from seven blank solutions.

The accuracy and precision of the cereal analysis were assessed using wheat (GBW08503a, national certified reference materials of China). Standard solutions were inserted into the sample sequence every 8 samples to verify sensitivity and repeatability. The recoveries of REE were 87.9–98.4%.

### Health risk assessment

The estimated daily intake of REE through cereal consumption was calculated by the following equations^36^:

$$EDI=\frac{Cv\ast CR}{BW}$$

where *EDI* (μg/kg bw per day) represents the estimated daily intake of REE, *Cv* is level of REE in cereal (μg/kg), *CR* is consumption rate (kg/day), *BW* is body weight (kg). For analytical results below the LOD, 1/2LOD was used to produce estimates.

Zhu *et al*.^37^ have proposed a daily allowable intake of 70 μg/kg bw for rare earth oxides, which was certificated from human health survey in REE mining areas and animal experimental results.

### Statistical analyses

Statistical analyses for comparing the average results of different cereal samplings were performed using Wilcoxon rank sum test. *P* < 0.05 was considered statistically significant. SAS 9.1.3 (SAS Institute Inc., Cary, N.C.) was used to conduct all of the analyses.

### Data Availability

The datasets generated during and/or analysed during the current study are available from the corresponding author on reasonable request.
